# Dietary factors associated with preeclampsia or eclampsia among women in delivery care services in Addis Ababa, Ethiopia: a case control study

**DOI:** 10.1186/s13104-018-3793-8

**Published:** 2018-10-01

**Authors:** Teklit Grum, Solomon Hintsa, Gebremedhin Hagos

**Affiliations:** 1 Department of Reproductive Health, College of Health Sciences, Aksum University, Aksum, Ethiopia; 2 Department of Epidemiology and Biostatistics, College of Health Sciences, Aksum University, Aksum, Ethiopia; 3 Department of Medical Science, College of Health Sciences, Aksum University, Aksum, Ethiopia

**Keywords:** Dietary factors, Preeclampsia or eclampsia, Women, Ethiopia

## Abstract

**Objective:**

Preeclampsia or eclampsia, which is one of the direct obstetric complication, results in maternal and child morbidity and mortality. The factors associated with it remains unclear. So, the aim of the study was to assess the dietary factors associated with preeclampsia or eclampsia among women in delivery care services in Addis Ababa, Ethiopia.

**Results:**

Factors which were investigated as protective for preeclampsia or eclampsia were: Fruit intake during pregnancy (AOR: 0.94, 95% CI 0.20, 4.32), vegetable intake during pregnancy (AOR: 0.95, 95% CI 0.01, 0.71) and receiving nutritional counseling during antenatal care (AOR: 0.17, 95% CI 0.05, 0.6). In the other side being nulliparous women was a risk factor for preeclampsia or eclampsia (AOR: 2.02, 95% CI 1.15, 3.55).

## Introduction

Preeclampsia is a syndrome characterized by hypertensive disorder and protein in the urine which usually occurs after 20 weeks of gestational age whereas eclampsia is the severest form of preeclampsia [1]. Its’ causes remains unclear and was responsible for many maternal and infants morbidity and mortality which accounts 5–10% worldwide [2]. Even though the magnitude of preeclampsia and eclampsia varies among countries, the burden is higher in developing than in developed countries [3].

In Ethiopia, preeclampsia and eclampsia is one of the major obstetric complications and was the most requirement for referral before onset of labour which accounts 83% [4].

It is obvious that pregnant women needs a healthy nutrition not only for the health of pregnant women but also for the child health [1]. Studies indicated that dietary habits are associated with preeclampsia and eclampsia [5–9]. Since, there are limited and inconsistence among the studies finding, this study will have its own contribution on assessment of factors associated with preeclampsia/eclampsia.

## Main text

### Study design

Facility based unmatched case control study was conducted.

### Study period and area

The study was conducted from December, 2015 G.C. to February, 2016 G.C. in two public hospitals in Addis Ababa, Ethiopia. Addis Ababa, where African Union is located, hosts an estimated 3.238 million people [10]. According to the city administration health bureau, the health coverage was 100%. Under the city administration there are 6 public hospitals and the study was conducted in 2 hospitals namely Zewditu Memorial Hospital and Ghandi Memorial Hospital.

### Inclusion criteria

Pregnant women with blood pressure of > 140/90 and presence of protein in urine was inclusion criteria for cases [1] and pregnant women who were not diagnosed for preeclampsia and eclampsia were controls. The inclusion criteria of gestational age for both groups were after 28 weeks.

### Exclusion criteria

Pregnant women with known hypertension and renal disease were excluded from the study.

### Sample size determination

Double population proportion formula was used to estimate the sample size using Epi-info 7 by considering the lowest adjusted odds ratio (2.34) and the proportion of exposed group in controls was (36.2%) from the Bahir Dar city study [6] which revealed unable to read and write was a risk factor for preeclampsia/eclampsia. During the sample size determination assumptions: 95% level of confidence, 5% marginal error and 80% power was considered. So, it was calculated to be 221 and after adding 10% for possible non-responses, the final sample size was estimated as 243 (81 cases and 162 controls).

### Sampling and data collection procedure

We select two public hospitals with largest delivery care load from the six hospitals under the city administration (Zewditu Memorial Hospital and Ghandi Memorial Hospital). Regarding to selection of the study subjects, all cases that diagnosed consecutively were included in the study. Once the case is diagnosed, two controls were selected immediately by simple random sampling technique and using delivery register as frame list.

Structured and pretested questionnaire was used to collect data by face to face interview. The questionnaire was initially prepared in English and translated to official language (Amharic). Four diploma holder midwifery were hired as data collectors and were supervised by two bachelor of sciences in midwifery. We use a quarter delivery report prior to study during allocation of proportional to size between the two hospitals. So, the Ghandi Memorial Hospital and Zewditu Memorial Hospital have quarter delivery report of 1496 and 1110 respectively. Accordingly, 138 (46 cases and 92 controls) study subjects from Ghandi Memorial Hospital and 105 (35 cases and 70 controls) form Zewditu Memorial Hospital were participated in the study.

### Data analysis procedure

The data was entered using Epi-info 7 and transferred to STATA 14 for further cleaning and analysis. Both Bivariable and multi variable analysis was done to see the association between the independent and dependent variables. Variance inflation factor (VIF) was checked for possible multi-colinearity among the variables. The model fitness for the logistic regression was checked using Hosmer and Lemeshow goodness of fit test statistics. Variables with p-value < 0.05 in the Bivariable analysis where included in the multivariable analysis to control confounding factors. Finally variables with p-value < 0.05 in the multivariable analysis was declared as a significant association with preeclampsia/eclampsia.

### Results

#### Socio-demographic characteristics of study participants

All participants (81 cases and 162 controls) respond to the study with response rate of 100%. The mean age of study participants was 27.22 years old with standard deviation of 5.03 and 81.07% of them belongs to 20–34 years age group. One hundred thirty-eight (56.79%) and 88 (36.21%) of the study subjects were orthodox followers and from Amhara ethnicity respectively. Concerning to educational status, only 24 (9.88%) of the participants had never attend formal education. The majority of the participants, 21 (82.72%) were married similarly 99 (40.74%) of the participants were housewife. The average monthly income of the study participants was 2921 Ethiopian Birr and nearly half of them had less than average (Table 1).

**Table 1:** Socio-demographic characteristics of women attending delivery care in Addis Ababa, Ethiopia 2016 G.C

Variables	Frequency	Percent
Age in years		
< 20	18	7.41
20–34	197	81.07
≥ 35	28	11.52
Mean age = 27.22 ± 5.03		
Religion		
Orthodox	138	56.79
Muslim	58	23.87
Protestant	30	12.35
Others	17	7
Ethnicity		
Amhara	88	36.21
Oromo	68	27.98
Gurage	36	14.81
Tigray	32	13.17
Others	19	7.8
Attending formal education		
Yes	219	90.12
No	24	9.88
Marital status		
Never married	21	8.64
Married or living together	201	82.72
Others	21	8.64
Occupation		
Housewife	99	40.74
Merchant	46	18.93
Employee	78	32.10
Student	20	8.23
Income (ETB)		
≤ 2921	118	48.56
> 2921	125	51.44

The variables checked for possible association with preeclampsia or eclampsia but were not significant in Bivariable analysis were; educational level, occupation, antenatal care follow up, history of abortion, history of stillbirth, cigarette smoking during pregnancy and chewing kchat during pregnancy. Having preeclampsia or eclampsia in previous pregnancy was significantly associated with preeclampsia or eclampsia in bivariate analysis (COR: 2.86, 95% CI 1.07, 7.65) but it remains insignificant in the multivariable analysis. Similarly, drinking coffee daily was associated with preeclampsia or eclampsia in bivariate analysis only (COR: 3.26, 95% CI 0.42, 25.36).

Receiving nutritional counseling during antenatal care follow up was found that protective for preeclampsia or eclampsia in the multivariable analysis (AOR: 0.17, 95% CI 0.05, 0.6). Being nulliparous was also associated with preeclampsia or eclampsia. The odds of developing preeclampsia or eclampsia in nulliparous women was 2.02 times higher than women with one or more parity (AOR: 2.02, 95% CI 1.15, 3.55). Regarding to pregnancy interval, women who had < 1 year pregnancy interval was at risk of developing preeclampsia or eclampsia comparing to women who had > 1 years pregnancy interval (AOR: 4.43, 95% CI 1.78, 11.04). Fruit and vegetable intake during pregnancy was a protective factor for preeclampsia or eclampsia independently. Comparing to women who didn’t eat fruit or vegetables, women who ate fruit or vegetable daily were less risk of developing preeclampsia or eclampsia (AOR: 0.94, 95% CI 0.20, 4.32) and (AOR: 0.95, 95% CI 0.01, 0.71) respectively (Table 2).

**Table 2:** Bivariable and multivariable analysis of women attending delivery care in Addis Ababa, Ethiopia 2016 G.C

Variables	Preeclampsia or eclampsia		COR (95%, CI)	AOR (95%, CI)
	Yes (%)	No (%)		
Previous preeclampsia				
No	26 (74.29)	91 (89.22)	1	1
Yes	9 (25.71)	11 (10.78)	2.86 (1.07, 7.65)	2.64 (0.77, 9.0)
Nutritional counseling during ANC				
No	32 (43.24)	17 (10.9)	1	1
Yes	42 (56.76)	139 (89.1)	0.16 (0.08, 0.32)	0.17 (0.05, 0.6)*
Parity				
Nulliparous	47 (58.02)	64 (39.51)	2.12 (1.23, 3.64)	2.02 (1.15, 3.55)*
≥ 1 parity	34 (41.98)	98 (60.49)	1	1
Pregnancy interval (year)				
< 1	13 (37.14)	12 (11.76)	6.56 (2.14, 20.06)	4.43 (1.78, 11.04)*
≥ 1	22 (62.86)	90 (88.24)	1	1
Fruit intake				
No	22 (27.16)	10 (6.17)	1	1
Daily	15 (18.52)	41 (25.31)	0.17 (0.06, 0.43)	0.94 (0.20, 4.32)*
At least once per week	44 (54.32)	111 (68.52)	0.18 (0.09, 0.41)	0.23 (0.06, 0.91)*
Vegetable intake				
No	15 (18.52)	5 (3.09)	1	1
Daily	6 (7.41)	35 (21.60)	0.57 (0.02, 0.22)	0.95 (0.01, 0.71)*
At least once per week	60 (74.07)	122 (75.31)	0.164 (0.06, 0.47)	0.25 (0.05, 1.37)
Coffee intake				
No	23 (28.40)	84 (51.85)	1	1
Daily	32 (39.51)	26 (16.05)	4.5 (2.25, 9.0)	3.26 (0.42, 25.36)
At least once per week	26 (32.10)	52 (32.1)	1.83 (0.95, 3.53)	5.09 (0.93, 27.97)

*COR* crude odds ratio, *AOR* adjusted odds ratio

* p-value < 0.05

### Discussion

We tried to investigate dietary intake during pregnancy factors associated with preeclampsia or eclampsia. As we expected, we found that fruit and vegetables intake during pregnancy as well as a healthy diet counseling during early antenatal care follow up were associated with reduction of preeclampsia or eclampsia. Besides, even though our primary aim was investigating dietary habits as a factor or not for preeclampsia or eclampsia development, we identified pregnancy interval with < 1 year and being nulliparous women as a risk factor for the outcome.

This finding revealed that nutritional counseling during pregnancy was associated with reduction of preeclampsia or eclampsia development. The odds of developing preeclampsia/eclampsia in pregnant women who took fruit daily during pregnancy were less comparing to pregnant women who didn’t take fruit during pregnancy (AOR: 0.94, 95% CI 0.20, 4.32).

During counseling on healthy diet, it addresses a wide range of nutritional values. For example, nutritional counseling on healthy dietary advice to consume vegetables and fruit benefit with regard to prevention of preeclampsia or eclampsia. Pregnancy is a period when most women are highly motivated for dietary advice. Because changes toward a healthier diet may also benefit their children [7, 9]. Dietary changes have a low cost, low risk compared with medical interventions, and have advantage beyond prevention of preeclampsia in public health importance.

Nulliparous women was at risk of having preeclampsia or eclampsia in this finding. Similarly a nested case–control study in Norway [11] investigated that nulliparous is a risk factor for preeclampsia. Unlike to this finding, a prospective study [12] indicates that being nulliparous is not a risk factor for preeclampsia.

Pregnancy interval was associated with preeclampsia or eclampsia. Pregnant women with < 1 year pregnancy interval was at higher risk of developing preeclampsia/eclampsia than pregnant women with > 1 pregnancy interval. In contrast, study on the assessment of risk factors made based on suggestive symptoms of preeclampsia, pregnancy interval was not associated with preeclampsia or eclampsia [13]. The inconsistency between the studies may be in the variation of preeclampsia diagnosis. Since the cases in that study was diagnosed by suggestive symptoms unlike to this study.

Fruit and vegetable intake during pregnancy was also found as protective factor for preeclampsia or eclampsia in this study. Fruit and vegetable dietary which are rich of vitamin C and vitamin E are associated with preeclampsia reduction. The prevention of these vitamins from preeclampsia development is due their anti-oxidant effects [9, 14].

The presence of high anti-oxidants in the plasma and placenta prevents hypo perfusion. As a result, the required level of these anti-oxidants enables endothelial cell to function normally. Following that hypertension and proteinuria, which are the clinical symptoms of preeclampsia, will not be developed [14].

### Conclusion and recommendation

This study mainly revealed that the role of dietary factors on the prevention of preeclampsia or eclampsia. Fruit and vegetable intake as well as receiving nutritional counseling during early pregnancy has a role on reduction of preeclampsia or eclampsia development. In another way being nulliparous women was identified as a risk factor for preeclampsia or eclampsia.

As recommendation, pregnant women should take fruit and vegetables starting from early pregnancy. Health providers should give nutritional counseling for pregnant women during antenatal care follow up. In addition to that, health providers should give focus for nulliparous while screening for preeclampsia or eclampsia.

## Limitations

As the limitation of the study, the amount of fruit and vegetable intake by pregnant women during pregnancy were not measured. In addition to that, even though preeclampsia or eclampsia can develop during post-partum period rarely, the cases were not followed after discharge.
